# Interdisciplinarity as cognitive integration: auditory verbal hallucinations as a case study

**DOI:** 10.1002/wcs.1305

**Published:** 2014-09-01

**Authors:** Marco Bernini, Angela Woods

**Affiliations:** 1 Department of English Studies, Durham University, Durham, UK; 2 Centre for Medical Humanities, Durham University, Durham, UK

## Abstract

In this article, we advocate a bottom-up direction for the methodological modeling of interdisciplinary research based on concrete interactions among individuals within interdisciplinary projects. Drawing on our experience in Hearing the Voice (a cross-disciplinary project on auditory verbal hallucinations running at Durham University), we focus on the dynamic if also problematic integration of cognitive science (neuroscience, cognitive psychology, and of mind), phenomenology, and humanistic disciplines (literature, narratology, history, and theology). We propose a new model for disciplinary integration which brings to the fore an under-investigated dynamic of interdisciplinary projects, namely their being processes of distributed cognition and cognitive integration.

## INTRODUCTION

In the last two decades interdisciplinary research has been pursued with increasing vigor within universities, centers, and laboratories all over the globe. However, methodological reflections on interdisciplinary research have not kept pace with this flourishing of cross-disciplinary projects and agendas, and the articulation of a transferable methodology for interdisciplinary work remains the ‘great challenge’ (Ref 1, p. 18). In this article, we advocate a bottom-up direction (derived *from* and accounting *for* the interaction of individuals) in methodological modeling. Instead of assessing potential intersections in the theoretical realms of each discipline we suggest that a model for cross-disciplinary work should be derived from (and embrace) concrete interactions among individuals within interdisciplinary projects. By drawing on our experience in a cross-disciplinary project on auditory verbal hallucinations (AVHs) running at Durham University, we focus on the dynamic if also problematic integration of the cognitive sciences (neuroscience, cognitive psychology, and philosophy of mind), phenomenology, and humanistic disciplines (literature, narratology, history, and theology). In the final section, we propose a new model for disciplinary integration which adds depth and complexity to otherwise flat, two-dimensional representations of disciplinary intersections. Importantly, this model brings to the fore an under-investigated dynamic of interdisciplinary projects, namely their being processes of distributed cognition and cognitive integration.^2^

## CONCEPTUALIZING INTERDISCIPLINARITY

Although in 1989 it was possible to dismiss interdisciplinarity as ‘the most seriously underthought critical, pedagogical, and institutional concept in the modern academy’ (Ref 3, p. 743), today it has a legitimate place in research practice,^4^ policy,^5^ and as an object of scholarly inquiry.^6-8^ While some have argued for the retention of broad, flexible, and even ‘slippery’ definitions of the term (Ref 6, p. 14) Julie Klein’s^9^ taxonomy outlines important distinctions between multi-, inter-, and trans-disciplinarity as follows: *multidisciplinary* approaches juxtapose knowledge, information, and methodologies from different disciplines in composite, sometimes collaborative, configurations (however, the disciplines ‘remain separate, disciplinary elements retain their original identity, and the existing structure of knowledge is not questioned’); *interdisciplinary* approaches emphasize integration as well as interaction, effecting disciplinary transformation at methodological as well as theoretical levels; finally, in *transdisciplinary* approaches, research questions and practices are framed by problems arising from the life-world and addressed by academics in partnership with other stakeholders.

Interdisciplinarity is a concept which is both fraught and fashionable (Ref 10, p. 255). Critics view interdisciplinary approaches as lacking in novelty, scholarly depth, and methodological rigor, and as espousing values and practices which serve the increasingly neo-liberal, market-driven agendas of the corporate University.^11^ Advocates argue that interdisciplinary approaches are essential to solving complex real-world problems such as climate change or poverty.^8,12^ In sharp opposition to those who would uncritically celebrate interdisciplinarity as the dismantling of disciplinary boundaries in the rapacious pursuit of new knowledge, Robert Frodeman has suggested that interdisciplinary research should be conceptualized in environmental terms as research which ‘recognizes limits—to people’s capacity for understanding, to time and money, and to research itself’ (Ref 13, p. 55).

The practical and institutional challenges of conducting interdisciplinary research are many, ranging from those located at the level of research funding and evaluation^12^ through to the training and motivation of individual researchers.^14-16^ It is increasingly recognized, however, that interdisciplinary case work (i.e., specific projects which tackle real-world problems) has an important role to play in advancing our capacity to understand and address these challenges from a ‘bottom-up’ perspective.^8,17^ For this reason we turn now to the analysis of an interdisciplinary project based at Durham University in the UK: Hearing the Voice (HtV).

## HTV: A CASE STUDY IN INTERDISCIPLINARITY

HtV is an ambitious interdisciplinary study of the phenomenon of voice-hearing, or AVH, funded by a 3-year Wellcome Trust Strategic Award (2012–2015, Charles Fernyhough, PI).*^a^* The core research team of 18 Durham-based academics, ranging from postgraduate to professorial level, have primary disciplinary affiliations in the arts, neuroscience, psychology, philosophy of mind, psychiatry, cultural studies, geography, history, literary studies and theology, and work with an extended research team of clinicians, mental health advocates, and people with lived experience of hearing voices. University support for the project in material as well as conceptual terms has been strong: the majority of academic researchers meet fortnightly at the Durham Institute of Advanced Study and work in offices co-located with the interdisciplinary Centre for Medical Humanities.

Despite the common misperception in clinical and cultural contexts that AVHs are inherently pathological (a hallmark symptom of schizophrenia, itself a deeply contested category^18^) their phenomenology is complex, heterogeneous, and not yet well-understood.^19,20^ AVHs are reported in nonclinical populations and by patients with a wide variety of diagnoses, they are measured and investigated using a range of empirical methods, as well as variously interpreted and valued in people’s lives across cultures and religious contexts, and as ‘talkative acts’ they are symptoms or experiences which are distinctively amenable to hermeneutic and linguistic analysis. For these reasons, HtV integrates scientific and humanities approaches to voice-hearing to achieve two high-level objectives. First, the project is pursuing multiple interdisciplinary lines of inquiry to attain a new holistic understanding of the phenomenon of voice-hearing, examining its significance as an aspect of personal narrative and as psychiatric symptom, conducting empirical studies into its cognitive and neuroscientific mechanisms, performing culturally sensitive investigations of its personal, social, and historical significance, and leading translational research into its therapeutic management. By drawing on and synthesizing multiple disciplinary perspectives and methodologies, the project seeks to move beyond the limitations inherent in approaching AVHs as discrete objects abstracted from the wider context of human experience or assigning biological psychiatry priority in their explanation. The second high-level objective is to develop a transferable methodology for interdisciplinary research into human experience which can be generalized to other areas of inquiry.

In addition to the project’s focus on AVH as a central if under-examined aspect of human experience, and explicit commitment to methodological reflection and innovation, the distinctiveness of HtV as a case study in interdisciplinarity can be identified in two further domains: recognition by the Wellcome Trust as a leading project in the relatively new field of the medical humanities,*^b^*^21^ and close engagement between the humanities and social sciences, clinical disciplines, and cognitive sciences.

## INTERDISCIPLINARITY AND THE COGNITIVE SCIENCES

If the historical separation of the humanities from the ‘hard’ sciences has been extensively theorized,^22,23^ the peculiar status of the cognitive sciences with respect to this division has only more recently come into focus.^24,25^ What distinguishes the cognitive sciences is not a reciprocal or even engagement with the humanities (if there is a ‘cognitive turn’ in the humanities,^26^ traces of a ‘humanistic turn’ in the cognitive sciences are, with few exceptions,^27^ absent), but rather their recruitment by disciplines such as literary studies,^28^ narratology,^29,30^ esthetics,^31^ or theology.^32^ While psychology, phenomenology, and philosophy of mind have exerted an influence on esthetic and cultural theory for over a century, only recently have experimental cognitive disciplines stimulated in the humanities such an array of enthusiasms, biases, and perplexities. Critiques of what has been termed ‘neuromania’^33^ are two-pronged. On the one hand, it is argued that the proliferation of ‘neuro-labels’ (neuroaesthetics, neurotheology, and cognitive narratology) is a fashionable trend and signals no substantial innovation on research hypotheses and outcomes that could have been framed or obtained by already existing disciplines. On the other hand, there is a suspicion concerning the methodological ground of these new ‘interdisciplines’. To what extent should neuroscientific methods inform humanistic research? Is this best conceptualized as a largely one-way interaction? And if not, how can the explanatory and interpretive toolkits of the humanities and social sciences modify the empirical and causal frameworks of cognitive sciences?

Among the supporters of the importance of the cognitive sciences for the esthetic field, Edward Slingerland^34^ has suggested that the cognitive sciences should provide a constraining function for humanistic research. This ‘vertical integration’—according to which hypotheses and method within the humanities should be vertically limited by what the cognitive sciences say can be a testable truth—clearly assigns a hierarchical priority to the scientific field. In what follows we aim to provide a different definition of disciplinary integration based on individuals’ interaction in the HtV project; a definition which can fully accommodate a mutual, more ‘horizontal’ exchange of methods and hypothesis between the cognitive sciences and humanities.

## FROM DISCIPLINARY INTERSECTION TO COGNITIVE INTEGRATION

### Missing Factors in Current Taxonomies

As we have seen, Klein’s^9^ taxonomy of interdisciplinary research assigns to multi- and interdisciplinarity a different degree of disciplinary involvement and plasticity (or malleability of disciplinary boundaries). Through juxtaposition and alignment, multidisciplinarity increases knowledge through encyclopedic additions that leave unaltered the contributing disciplines. The distinguishing feature of a strong interdisciplinarity is instead the proactive processes of *interaction* and *integration*. When deep interdisciplinary takes place we have, in Burns’ terms (Ref 35, pp. 11–12), an explicit ‘focusing’ and ‘blending’ of approaches in which new questions and/or methods emerge that were previously not belonging to individual disciplines. As Klein explains, integration can be partial or full, and its focus narrow (as in between ‘disciplines with compatible methods, paradigms, and epistemologies such as history or literature’) or broad, as in cases where there is little or no compatibility (Ref 9, p. 18).

By bringing together the cognitive sciences, phenomenology, and the humanities, HtV exemplifies strong integration with a broad interdisciplinary focus. However, in what follows we use the case study of HtV to highlight aspects of interdisciplinarity which are not accounted for by these taxonomies. On the one hand, we want to counter the sort of static description of interdisciplinary encounters, in which the degree of interaction seems to be pre-established and unchanging (time factor). On the other hand, we want to advocate the importance of considering interdisciplinarity not (only) as a disembodied interaction of disciplines on a theoretical level, but as concrete processes involving individuals’ entanglements in space and time (extended, embodied, and enactive factors). To put it succinctly, what is missing from Klein’s categorization is a cognitive description of interdisciplinary research. We suggest that looking at what cognitive sciences and cognitively informed phenomenology say about distributed cognition can and should profitably inform models of interdisciplinary research, complementing sociological and ethnographic approaches to the study of actors and agents in contexts of scientific investigation.^36-39^ In so doing, we propose to complement the current idea of strong interdisciplinary integration and interaction with a view of interdisciplinarity as a social form of what Richard Menary has labeled ‘cognitive integration’.^2^

### The Limits of Intersection (Time Factor)

**FIGURE 1:**
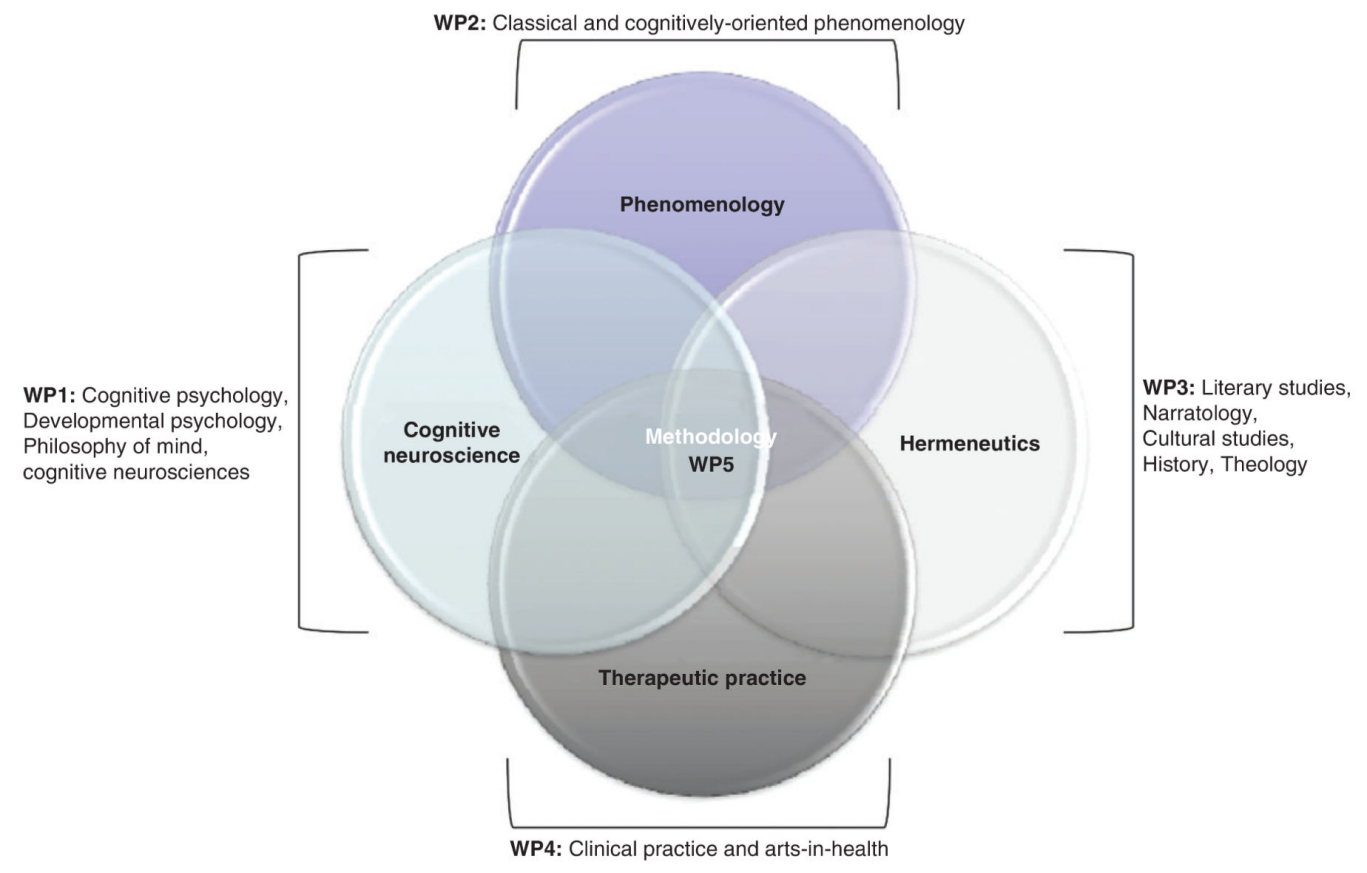
Hearing the Voice: A Venn diagram showing intersections between the five project workpackages.

At the beginning of our project we started by visualizing the project’s four largely discipline-based and one methodological workpackage through a classic Venn diagram (Figure 1).

This diagram has proven to be an effective heuristic tool for a preliminary project planning, and for communicating key areas of research to funders and others stakeholders. Yet, after the first year of the project, its limitations have become clear. The Venn diagram has its historical and disciplinary roots in the mathematical branch of set theory. Loosely speaking, its function is to display logical intersections between classes (finite sets) of objects; its explanatory potential therefore resides in assessing possible (but already existing) relations between them. The problem in using Venn diagrams for the description of interdisciplinary projects is twofold: first, disciplines are not finite sets but historically evolving organisms with uncertain boundaries; related to this is presentation of relations between them as fixed and static in time. To the extent that disciplines are not simply aligned, but not yet dynamically integrating and interacting, the Venn diagram appears to describe, in Klein’s taxonomy, something in between multidisciplinarity and interdisciplinarity. The plasticity of each discipline, which is a necessary condition for a proper integration, requires and occurs in time, a factor that is completely absent from this model of intersection. In the case of HtV, the Venn diagram is unable to capture the disciplinary interrogation and (re)definition, conceptual exchanges, emergent alliances, and problematic frictions of the project’s first year. The time factor in disciplinary plasticity is not the only dynamic aspect missing from intersecting and taxonomical models. Relatedly and, we argue, more importantly, disciplines are always considered as disembodied entities with definite boundaries. Our view is that dynamic processes of integration and interaction must take account of the fact that individuals are the bearers and beholders of knowledge and methods. Prioritizing the embodied and social components of interdisciplinarity projects, we propose an account of disciplinary encounters in terms of extended^40,41^ and enactive^42,43^ cognition.

### The Limits of Theoretical Integration (Extended, Embodied, and Enactive Factors)

In referring to the mutual ‘cannibalization’ of theories and their unsystematic incorporation of sometimes conflicting claims, Jacques Derrida defined theories as ‘monsters’.^44^ This monstrosity can be detected also in disciplinary umbrella terms such as ‘literary studies’, ‘medical humanities’, and ‘cognitive sciences’. So varied are its contributing disciplines, and so heterogeneous are its approaches, that to treat the field of ‘cognitive sciences’ as a unitary agent in the interdisciplinary exchange is problematic, to say the least. The habit of speaking of disciplines as unified agents relates to our functional tendency to simplify and to anthropomorphize. But if disciplines are not individual, they are embodied in (and their existence and development rely on) individual human agents. Instead of approaching interdisciplinarity in the abstract terms of a theoretical integration, we propose to ground analysis in the actual interaction of what we can call individuals’ *disciplinary minds*. This methodological turnaround, from a top-down categorization of interdisciplinarity to a bottom-up account, complements formulations of disciplinary actors and agents within science and technology studies, and has two important benefits. First, it reconceptualizes the abstract problem of disciplinary boundaries in terms of the boundaries of human minds and in terms thus amenable to cognitive inquiry. Secondly, by drawing on contemporary accounts of extended, embodied, and enactive cognition, it enables us to understand the ‘unbounding’^2^ of disciplinary minds in interdisciplinary projects.

Cognitive sciences now strongly challenge the Cartesian idea that cognition is something that happens *just* ‘in the head’.^45^ According to this new framework in the science of the mind,^46^ cognitive processes are instead partially extending into the world through the interaction with the environment and cognitive tools.^40^ The extended mind (EM) thesis assumes that the boundaries of the mind (the conceptual edges underlying the opposition of internal versus external space) become under certain conditions ‘porous’.^47^ This porosity takes place when the mind interacts, manipulates and exploits external tools; when the mind couples with these externalities and constituting what EM theorists call a ‘coupled system’. By drawing on recent expansions of this thesis into the social domain,^48,49^ in the next section, we want to suggest that, once we consider interdisciplinarity as the embodied and enactive interaction of individuals (shortly, as a human cognitive process) the same ‘porosity’ is activated in what we called ‘disciplinary minds’. Furthermore, we propose a model that accommodates the same causal reciprocity and agency distribution between disciplinary minds that the EM thesis attributes to the mind–world interaction.

## BLUEPRINT FOR A WE-SPACE: TOWARD A CROSS-DISCIPLINARY COGNITIVE INTEGRATION

Two main principles guide the EM thesis, according to which the mind extends into the world by interacting with cognitive tools. The first concerns the hierarchical cognitive relation of the two poles involved in the interaction and it is referred to as the ‘parity principle’. This principle implies that both poles have an equal cognitive status, for when the mind is coupled with a part of the world ‘that part of the world *is* (so we claim) part of the cognitive process’ (Ref 45, p. 29). The second principle is about the causal functioning within a coupled system, which is described as a ‘continuous reciprocal causation’ (CRC). This principle is about reciprocity between the parts, and it occurs ‘when some system *S* is both continuously *affecting* and simultaneously *being affected* by, activity in some other system *O*’.^40^ To put the two principles together, when the mind interacts with specific externalities couple systems are activated, and within these systems a mutual horizontal two-ways affection is generated. Neither of the parts (the mind and the worldly component) remains untouched due to the activation of ‘inextricable tangles of feedback, feedforward, and feedaround loops that promiscuously criss-cross the boundaries of brain, body, and the world’ (Ref 47, p. 277). In terms of cognitive enhancement of thinking and performing, this extension, the EM claims, ‘make possible or *fosters* forms of thought which were *previously difficult or impossible*’ (Ref 2, p. 622 and Ref 50, p. 103).

**FIGURE 2:**
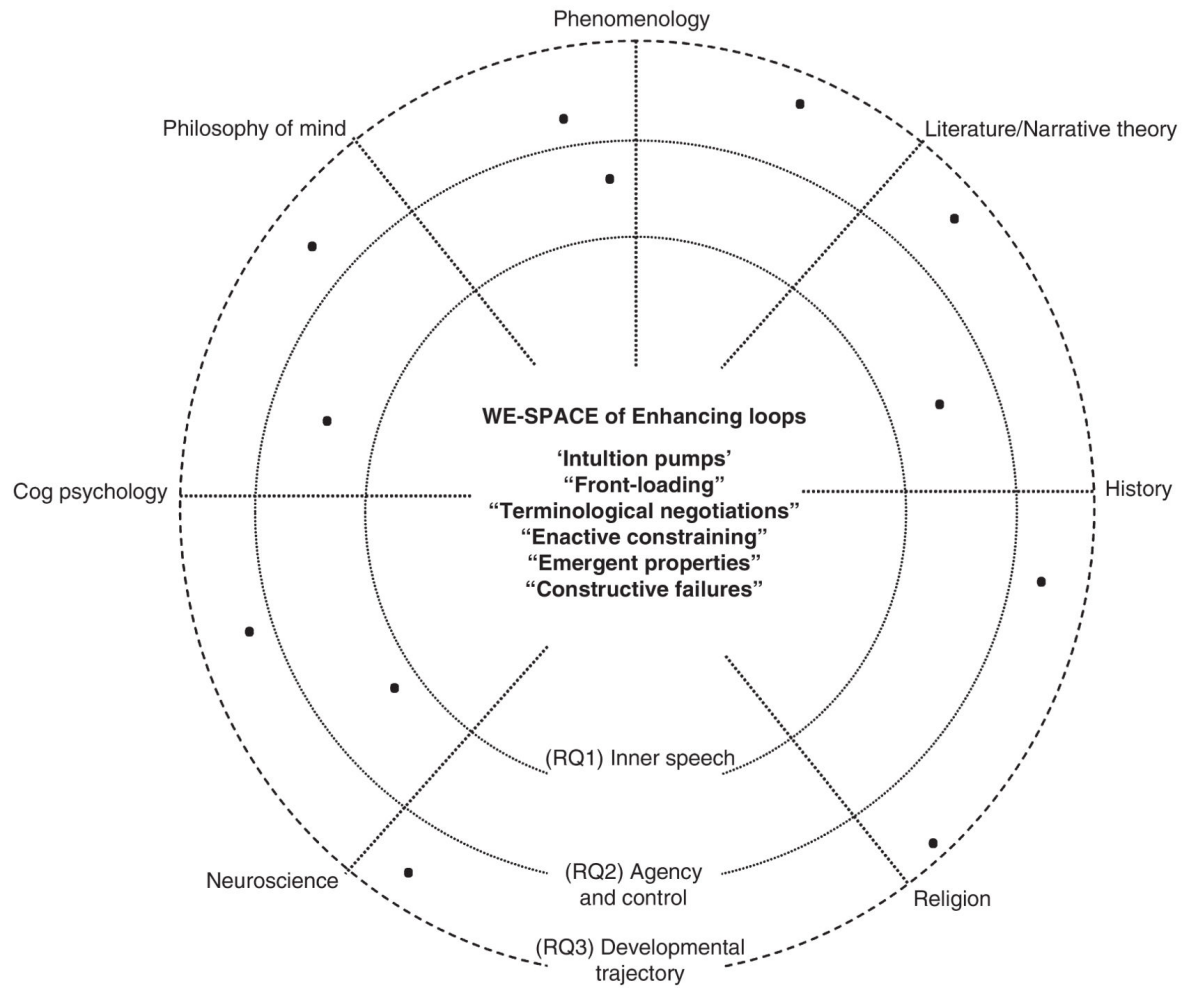
Blueprint of Hearing the Voice. With the contributing disciplines in the angular dimensions and different research questions in the radial axes. (Adapted and modified from Varela, Thompson and Rosch 1991)

EM thesis explicitly limits the rank of cognitive tools enabling this activation of loops to technologies (broadly intended, from computers to writing), but recently this thesis has been tentatively applied also to social cognition—an application that appears particularly warranted if viewed in relation to earlier social theories of cognition such as Vygotsky’s.^51-53^ For instance, Shaun Gallagher’s idea of a *socially* EM ‘builds on the enactive idea of social affordances. Just as a notebook or a hand-piece of technology may be viewed as affording a way to enhance or extend our mental possibilities, so our encounters with others, especially in the context of various institutional procedures and social practices may structures that support and extend our cognitive abilities’.^48^ Similarly, Joel Krueger has argued that ‘social cognition is a kind of extended cognition’, and that it ‘is fundamentally an interactive form of space management—the negotiation and management of a “we-space”’ (Ref 49, p. 643). We think this shift regarding a social application of the EM’s tenets provides solid ground for a further tweak, i.e., the application of the EM to the social interaction of disciplinary minds. By adapting a model, Varela, Thompson, and Rosch used to visualize the different approaches within cognitive sciences in their foundational work about enactive cognition.^42^ We propose the following model as a better account of interdisciplinary enhancing loops and cognitive integration in the HtV project (Figure 2).

We called this a ‘blueprint model’ because it can *actually* be transformed into a physical building. Even without this concretizing operation, however, the model is not entirely conceptual and metaphorical. In HtV, we *do* have a central meeting space, a social ‘we-space’ constituted by a physical room where we have our fortnight meetings (‘Voice Club’) structured and facilitated by artist Mary Robson. The concentric circles emanating from this physical ‘we-space’ are the ever-changing research questions generated within it. Touching on every discipline in the project, these questions open up specific, temporally contingent spaces in which individual researchers interact. The model accommodates two temporal dimensions: synchronic entanglements (in the immediate ‘we-space’ of ‘Voice Club’), as well as the diachronic or longer-term entanglements emerging from prolonged engagements as participants move together in a common problem space, slowly adjusting to—and integrating with—other disciplines’ ways of thinking, speaking, and framing problems.*^c^*

Two criteria guide the disposition of disciplines in the angular dimensions—read clockwise they show (1) a contiguity of methods and research topics and (2) a major toward minor reliance on empirical experimental data. In this respect, phenomenology is significantly a boundary discipline between the scientific and humanistic semi-circles. The radial axes are generated by different research questions and hence change throughout the project. This model could be easily be manipulated and modified to accommodate a larger or smaller numbers of disciplines and research questions, but our illustration focuses on three aspects of AVH to which all disciplines are called to contribute: (RQ1) the relationship between inner speech and AVHs (discussed in more detail below); (RQ2) the degree to which voice-hearers’ recognize their voices as agents over which they have various degrees of control; and (RQ3) the developmental trajectory of AVH across the lifespan. Black dots are people involved in the project, or what we have called ‘disciplinary minds’. The model makes clear that people are not just positioned by their main discipline of expertise, but can be located in a greater or less proximity to the contiguous field. Furthermore, along the project their location can vary both in the angular dimension and radial axis, following their role in the new research questions (that can be virtually endlessly expanded) and their disciplinary polarization for those specific topics. Another important aspect to underline is that the concentric circles are all dashed, emphasizing movement and exchange including with actors ‘outside’ the project, and avoiding a hierarchical orientation of the model in terms of center versus periphery.

Although subgroups form and interact throughout the project space, the central physical we-space of ‘Voice Club’ offers the starkest presentation of the interdisciplinary project as a cognitively integrated system of extended disciplinary minds. This is the space in which *all* the disciplinary minds interact; interactions which activate what we might call ‘enhancing loops’ (feedback loops which produce cognitive enhancement, i.e., they disclose theoretical and/or testable hypotheses previously unthinkable by a single disciplinary mind) These include:

- *Intuition Pumps*: In Dennett’s terms, intuition pumps are tools which stimulate specific kinds of thinking. These ‘imagination extenders’ and ‘focus holders’ (Ref 54, p. 2) are present in each discipline (and disciplinary mind), from the philosophical Plato’s cave to the literary Aesop’s short stories. In disciplinary cognitive integration, though, new intuition pumps can be generated and previous pumpers empowered or manipulated by interacting with other disciplinary minds.
- *Front Loading*: Shaun Gallagher’s^55^ call for insights from phenomenology to be ‘front-loaded’ into the design of cognitive sciences experiments need not be limited to a single discipline, as we have argued elsewhere.^20^ In the case of HtV, what to load and how has been the subject of two ‘neurohackathons’ in which humanities researchers have participated directly in experimental design. Furthermore, front-loading should not be considered a one-way process. In looping, each discipline can be loaded by and load insights that will be mutually affecting.
- *Terminological Negotiations*: Each discipline has different words for describing a similar concept, or the same word signifying completely different meanings. The negotiation of terminology, however, is more than looking for existing intersection. As with all the processes we are describing, this can generate loops leading to unexpected terminological and conceptual innovations.
- *Enactive Constraining*: Interdisciplinarity is not an intellectual performance, an application of knowledge. In the enactivist terms, is not a form of ‘knowing-that’ (propositional knowledge) but a form of ‘know-how’ (enactive exploration).^56^ Disciplinary minds discover in the inter*action* and extension with other minds what is possible to *do* and what is not. There is a mutual constraining or, in Gallagher’s words, each disciplinary mind provides a specific and limited kind of affordance.
- *Emergent Properties*: The phenomenon to be investigated and explored by interdisciplinary projects (in our case, AVHs) has probably already distinct features and properties in many discipline involved. The coupled interdisciplinary extension of disciplinary minds allows the emergence of unpredicted (and, according to the novelty typical of a proper cognitive integration, *unpredictable*) properties.
- *Constructive Failures*: Failures are rarely in the spotlight of interdisciplinary debate, but we regard them as telling signs of *resisting integrations*. Understanding why feedbacks loops are not generated or the reasons for the impossibility of front-loading experimental designs is a constructive form of enactive disciplinary exploration. These failures, in fact, affect further interactions as well as successful integrations.

This is a partial list of the kind of activities creating/generated by enhancing loops within the project. Among the several examples we are collecting throughout the project, we can briefly refer to the interaction of disciplinary minds in addressing (RQ1) the relationship between inner speech and AVHs. Together with the formal presentation of existing research in psychology and philosophy,^57-59^ we have conducted close reading and narratological analyses of the varieties of inner speech in literary texts by Samuel Beckett and Hilary Mantel; in-depth discussion in small groups of the scales and measures used to investigate inner experience, and the subsequent development and implementation of new tools for phenomenological research; and simulation of the meditative practices used by monks in the middle ages to quiet turbulent inner voices. We have also used these investigations to cast, confront and reciprocally constrain hypotheses for empirical research (phenomenological questionnaires and fMRI studies) While the findings and quantifiable outputs of many these entanglements (which began in late 2012) have yet to be published, their effects are evident in the questions and directions now being explored by researchers within the project, as well as in the qualitative data gathered through formal and informal evaluations of ‘Voice Club’.

Although not an exhaustive list, the enhancing loops at operation within HtV all function to produce thoughts and outcomes that, as the EM thesis indicates, were *unthinkable* or *unpredictable* before, even if subtly so. All these cognitive practices between disciplinary minds follow a horizontal ‘parity principle’ and loops of ‘CRC’; every interaction is at the same time a coupled system in itself and part of the larger cognitive system, which is the project as a whole. The model, then, allows us to trace the holistic understanding of a phenomenon at issue (AVHs) by portraying *the cognitive unity of the project as a whole* as well as the small-scale integrated and integrating couple systems constituted by individuals’ interactions. It also allows us to grasp the complexity of potential what Fitzgerald and Callard have termed ‘experimental entanglements’.^60^ ‘To be entangled’, they write, ‘is precisely not simply to labour together, or to compare - or engage in “dialogue” about – our different disciplinary perspectives. It is to proceed, instead, on the assumption that entanglements – of bodies, epistemologies, apparatuses, elements of experimental systems, operationalizations of terms – might produce something new in the world, even as the forms that that newness might take are undecided, and undecidable, prior to the moments of experimentation’.^60^

## CONCLUSION

Whether in Kant’s call to view every science ‘as a separate and independent building … a self-subsisting whole’ (Ref 61, p. 31), Becher’s discussion of disciplinary incursion as hostile colonial encounter,^62^ or Docherty’s fear that interdisciplinarity lets ‘our disciplines overflow into each other like anarchic lava lamps’^63^ spatial and particularly architectural metaphors have dominated the conceptualization of disciplinary distinction and interdisciplinary integration. In this article, we offered an account of interdisciplinarity as a form of cognitive integration grounded in an analysis of the HtV project. The model (or spatial metaphor) we proposed allows us to (1) move from an idea of ‘vertical integration’ to horizontal nonhierarchical interactions; (2) reintegrate the extended, embodied, and enactive factors of interdisciplinarity by focusing on individual disciplinary agents; (3) treat interdisciplinary as cognitive process of socially extended ‘disciplinary minds’; (4) better represent the dynamic temporal and spatial dimensions of interdisciplinary research (the position and relation of disciplinary minds will change with each new research question). In essence, the model puts the onus on the cognitive dynamics among individuals engaged in interdisciplinary projects, and therefore can be consistently applied also to other projects even when cognitive sciences are not involved.

We believe that this cognitive account of interdisciplinarity has great potential for further development in at least two directions. First, we have not accounted here for the emotional dimensions of social cognition, or the training, motivation, skills, attitudes, and even ‘virtues’ (Ref 13, p. 34) which play a role in interdisciplinary research. Second, our model does not yet capture the transdisciplinary modes of engagement that characterize critical medical humanities research. What new enhancing loops are created when nonacademic partners—clinicians and voice-hearers—enter and navigate these spaces? What happens when disciplinary minds venture into undisciplined territories outside the academy^13^? Shifting the debate on interdisciplinarity from a disembodied abstract level to an account of its extended, embodied, and enactive processes discloses a complexity which calls for more sophisticated models. Our article provides the ground for future advances in this direction.

## Footnotes

a Further details are available from the project web site: https://www.dur.ac.uk/hearingthevoice/.

b Tracing its roots to medical education and bioethics, the medical humanities has traditionally referred to the use of ideas and methods from the arts and humanities to ‘rehumanise’ healthcare. More recently, and particularly in the UK, the idea that the humanities are supplementary, but ultimately peripheral to medicine has been challenged, and the medical humanities have developed into a vibrant interdisciplinary research field exploring the social, historical, and cultural dimensions of scientific knowledge, clinical practice, patient experience, and health policy. As the first medical humanities project to receive Strategic Award funding in open competition with the biomedical sciences, the HtV project can be seen to reflect emerging perceptions of what the Wellcome Trust regard as cutting-edge research in this field, namely (1) atransdisciplinary engagement with patients and clinicians, which is predicated on (2) a robust commitment to broad interdisciplinarity (reciprocal exchange and integration between disciplines with little recognized compatibility, like medieval history and cognitive neuroscience).

c We are grateful to Joel Krueger for this insight.
